# Inner versus Overt Speech Production: Does This Make a Difference in the Developing Brain?

**DOI:** 10.3390/brainsci10120939

**Published:** 2020-12-05

**Authors:** Franziska Stephan, Henrik Saalbach, Sonja Rossi

**Affiliations:** 1 Department of Educational Psychology, Faculty of Education, University Leipzig, 04109 Leipzig, Germany; henrik.saalbach@uni-leipzig.de; 2 Leipzig Research Center for Early Child Development, 04109 Leipzig, Germany; 3 ICONE, Innsbruck Cognitive Neuroscience, Department for Hearing, Speech, and Voice Disorders, Medical University of Innsbruck, 6020 Innsbruck, Austria

**Keywords:** inner speech production, overt speech production, event-related brain potentials (ERPs), functional near-infrared spectroscopy (fNIRS), preparation of speech production

## Abstract

Studies in adults showed differential neural processing between overt and inner speech. So far, it is unclear whether inner and overt speech are processed differentially in children. The present study examines the pre-activation of the speech network in order to disentangle domain-general executive control from linguistic control of inner and overt speech production in 6- to 7-year-olds by simultaneously applying electroencephalography (EEG) and functional near-infrared spectroscopy (fNIRS). Children underwent a picture-naming task in which the pure preparation of a subsequent speech production and the actual execution of speech can be differentiated. The preparation phase does not represent speech per se but it resembles the setting up of the language production network. Only the fNIRS revealed a larger activation for overt, compared to inner, speech over bilateral prefrontal to parietal regions during the preparation phase. Findings suggest that the children’s brain can prepare the subsequent speech production. The preparation for overt and inner speech requires different domain-general executive control. In contrast to adults, the children’s brain did not show differences between inner and overt speech when a concrete linguistic content occurs and a concrete execution is required. This might indicate that domain-specific executive control processes are still under development.

## 1. Introduction

When having to self-regulate cognition and actions, speech is an important mediator during childhood and adulthood [1,2]. Speech not only relies on spoken output (overt speech) but also on the silent production of language (inner speech). Life-span theories suggest that the use of inner speech undergoes a maturational change from early to middle childhood until adolescence [3,4]. The very early development of inner speech results from social interactions between children and caregivers, mostly in an overt manner. Afterwards, overt speech transforms such that children speak aloud to oneself. Subsequently, at around 6–9 years, overt speech becomes more internalized being gradually transformed into inner speech with some external manifestations like whispering, muttering, or lip moving [1,5,6,7,8]. Therefore, the primary school age is a promising time window to investigate the maturation of the internalization process of language. This internalization process is strongly associated with maturing cognitive abilities. A number of behavioral studies have come to the conclusion that the more speech is internalized to inner speech, the better children performed on several cognitive tasks and the better their academic achievements were [6,8,9,10,11,12,13,14]. One difficulty with the assessment of inner speech by behavioral approaches is that children who do not speak so much overtly might, as a consequence, use more inner speech, but they might also be simply more introverted. This results in difficulties to directly assess inner speech as no apparent speech output occurs [1]. Thus, the present study aimed at investigating neural mechanisms of inner and overt speech in children. Assessing brain activity provides the advantage of directly assessing differences or similarities without a concrete speech reaction from the child. We examine the fine-grained temporal dynamics and brain areas underlying inner and overt speech processing in children by the simultaneous application of electroencephalography (EEG) and functional near-infrared spectroscopy (fNIRS) [15] during a picture-naming paradigm [16].

### 1.1. Overt Versus Inner Speech Execution

There is an ongoing debate about the question of whether overt and inner speech are similar (unimpoverished hypothesis) or whether inner speech is attenuated at phonological and phonetic levels (surface-impoverished hypothesis) [17,18,19,20,21]. Derived from a speech production model [22], these hypotheses share the view that overt speech production entails multiple consecutive processing steps. This view was supported by evidence from neuroscientific methods over the last decades [23,24,25,26,27,28]. Speech production processing is assumed to start with the conceptual preparation, lemma retrieval and lemma selection until about 275 ms after stimulus onset, supported by middle temporal regions, followed by phonological code retrieval supported by middle and superior temporal regions. The syllabification (phonological encoding) starts at around 355 ms in frontal regions. The subsequent phonetic encoding starts at around 455 ms in frontal, predominantly motor-related areas, and is followed by the articulation process starting at about 600 ms after stimulus presentation. In a recent study, inner and overt speech in adults were compared by simultaneously using EEG and fNIRS [16]. Findings showed some evidence for the surface-impoverished hypothesis. In particular, EEG results during speech production (i.e., the execution) showed a larger negativity, reflecting inhibitory (i.e., linguistic control) processes for inner compared to overt speech between 300–500 ms. This time range corresponds to the stage of phonological code retrieval and thus suggests similar lexical but different phonological processes. This resembles an impoverishment of inner speech at the phonological level [29,30,31,32]. fNIRS results showed a larger activation for overt compared to inner speech in bilateral temporal regions during speech execution [16]. This points towards the involvement of auditory feedback processes after listening to the produced speech output. However, is the surface-impoverished hypothesis also valid for children? To our knowledge, neuroscientific studies directly comparing inner and overt speech in children do not exist. Neuroimaging studies investigating overt language production in children and adults found largely overlapping activated brain regions between these two groups but with different activation strengths [33,34,35,36]. These differences in activation between children and adults were assumed to reflect functional changes in some brain regions across age. In the same line of reasoning, some EEG studies investigating overt speech in children found the same event-related brain potential (ERP) components as in adults but with differences in latency and amplitude [37,38,39]. However, these results were found for specific ERP components in various experimental designs and at different ages. Thus, it is hardly possible to conclude whether all speech production steps or only specific processes correspond to those of adults. A study of Laganaro et al. [40], trying to solve this issue, investigated the temporal dynamics of overt speech production in school-age children (7 to 8 years and 10 to 12 years) compared to adults performing a picture-naming task. The results showed that children displayed latency shifts and larger amplitudes of early ERP components, mainly related to immature visuo-conceptual processes. Furthermore, lexical retrieval processes were also delayed in children while later phonological and phonetic encoding did not vary between children and adults. The authors suggested that differences in latencies reflect different degrees of efficiency, in particular for lexical processes from childhood to adulthood. Nevertheless, it remains open how exactly inner and overt speech and potential impact of control processes differ in the children’s brain when inner speech is still under development [1,5,6,7,8].

### 1.2. Overt Versus Inner Speech Preparation

Each motor act, such as the execution of speech, must be adequately prepared. Stephan et al. [16] used a design created by Gehrig et al. [41] as well as Kell et al. [42] in which the pure preparation of a subsequent speech production (preparation phase) and the actual execution of speech (execution phase) can be differentiated. In the preparation phase, participants only received information about how to produce the stimuli (either aloud or silently) during the subsequent execution phase. The preparation phase gives the opportunity to examine whether domain-general executive control processes are similarly active when inner and overt speech have to be prepared for subsequent speech production [41]. Because the preparation phase is free of linguistic and motor processes, it enables the disentanglement of domain-general from more linguistically driven executive control mechanisms. Specifically, the preparation phase does not represent speech per se but it resembles the setting up of the language production network. Gehrig et al. [41] as well as Kell et al. [42] in this regard refer to domain-general executive control as a mechanism that controls the selection of rules and afterwards implements the selected rule into goal-directed behavior. Thus, the investigation of the preparation of language production allows the examination of preparatory processes, which precede the linguistic processing (i.e., the specific lexical and phonological retrieval and encoding), motor planning, and execution of articulation. Interestingly, the EEG and fNIRS findings of Stephan et al. [16] demonstrate that the adult brain already differentiates between inner and overt speech during the preparation phase when no linguistic content or motor processes are involved, suggesting different executive control processes for overt and inner speech, because overt and inner speech elicit different sensory and motor consequences of speaking [16]. Thus, using a preparation phase allows the examination of executive control processes involved in setting up the language network system. In general, language production engages domain-general (nonlinguistic) executive control such as inhibition, attention, and goal-directed behavior [41,42,43,44] as well as domain-specific (linguistic) executive control such as lexical, phonological and syntactical processes [23,45,46]. In particular, nonlinguistic as well as linguistic executive control act together during the execution of speech. By investigating the preparation and execution of language production in children we aim to examine the pre-activation of the language network in order to disentangle domain-general executive control processes from linguistic control (domain-specific, i.e., concrete lexical and phonological retrieval and encoding) in the developing brain.

### 1.3. The Present Study

**Figure 1:**
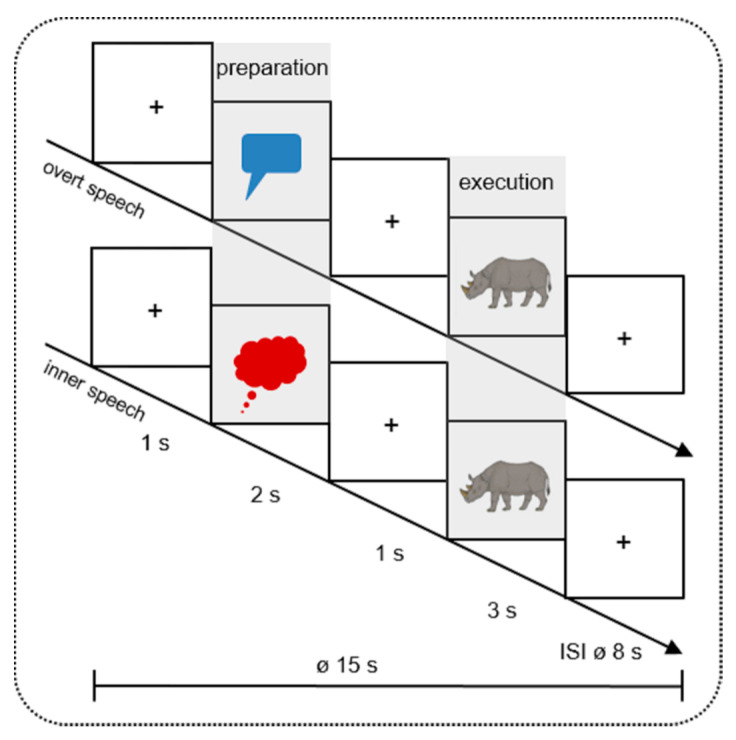
Design of the study. In 16 mini-blocks, 40 different colored pictures were presented twice (in inner and overt speech condition). Every mini-block contained 5 trials of the same condition. During the Preparation Phase, the pictures were cued by a red speech (overt speech condition) or a blue thinking (inner speech condition) bubble. During the Execution Phase, participants had to name the pictures (e.g., the rhinoceros) aloud or silently. The pictures were taken from Rossion and Pourtois [49] with images courtesy of the authors. + indicates a fixation cross; ø indicates the mean duration of each trial (15 s) and inter-stimulus-interval (ISI 8 s).

Nevertheless, it remains open how exactly inner and overt speech are processed in time and how the preparatory activity guides the subsequent speech execution in children. To address this issue, the present study used a design similar to the previously mentioned studies [16,41,42] by comparing an actual speech execution phase with a speech preparation phase (see Figure 1). In order to identify temporal and topographical processes, EEG and fNIRS were applied simultaneously. The former provides an exquisite temporal resolution whereas the latter bears the potential to identify involved brain regions. Both methods are ideal to investigate language production in children [15,47,48].

The main research question under investigation is whether similar or different processing steps are present for inner and overt speech during the preparation and execution phase in children and adults, suggesting a comparable or contrasting involvement of executive control, linguistic and motor processes. In particular, we addressed these research questions:

(1) Do inner and overt speech differ topographically and temporally during the preparation phase? Although there is evidence in adults that, before speech is acted out, the brain controls for the sensory and motor consequences of speaking [16,41,42], there exists no evidence on whether the child’s brain is able to set up this language network and whether there are differences in the use of executive control in overt and inner speech.

(2) Do inner and overt speech differ topographically and temporally during the execution phase? We aim at investigating the surface-impoverished and unimpoverished hypothesis in children. If the surface-impoverished hypothesis also applies to children, a differential processing of inner and overt speech during phonological code retrieval and encoding (around 300 ms) as well as frontal and/or temporal regions is expected during the execution phase. If, however, no such difference can be attested, this would speak for the unimpoverished hypothesis in children. This might be the case considering behavioral studies showing that inner speech production in children around 6–9 years can be accompanied with external manifestations like whispering, muttering, or lip moving implicating activation of phonological and phonetic processes during inner speech [1,5,6,7,8].

## 2. Materials and Methods

### 2.1. Participants

29 healthy native German speaking children (13 females; mean age: 6.52 ± SD 0.51; age range: 6 to 7 years) completed the study. All participants were first-grade pupils. Many studies have shown that in this age children are just about to start to internalize overt speech for self-regulation purposes. So although they have a sense of inner speech, they are still in the process of internalization [6,7,8]. Inclusion criteria were the following: right-handed (assessed by means of the Edinburgh Handedness Inventory [50], lateralization quotient: 86.32 ± SD 3.59; range: 40–100), normal or corrected-to-normal vision, no bilingualism, no prematurity, and no hearing, language, or neurological disorders. All participants were included in the same EEG and fNIRS analysis cohort.

Prior to the experiment the three-item questionnaire of Flavell et al. [51] was conducted to assess the awareness of inner speech in order to ensure that children were able to fulfil the inner speech picture naming task (e.g., “Can a person tell himself things or talk to himself up in his head?”). Moreover, children were asked one further question developed by the authors, “Can you tell yourself things or talk to yourself up in your head?”, to assess whether children know about their own inner speech. If the children would have negated at least one of the questions, self-awareness training would have been conducted. In our cohort no child necessitated this training.

To ensure typical vocabulary development, children’s expressive vocabulary abilities were assessed with the language development test for children aged 5–10 years (SET; Sprachstanderhebungstest für Kinder im Alter zwischen 5 und 10 Jahren) [52] using the subtest 1. The subtest consists of 40 pictures the children had to name. According to this test the vocabulary of all participants corresponded to the chronological age of the children and was thus in the normal range (T-values: 68.52 ± SD 1.56; range: 51–80).

### 2.2. Materials

To ensure a feasible task for children we used a single-word picture-naming paradigm. The stimulus material consisted of 40 colored drawings selected from the revised standardized set of Snodgrass and Vanderwart [53] by Rossion and Pourtois [49], by means of a rating performed by 20 adults (16 females, mean age 27.7 ± SD 5.79). The aim of the rating was to assess the pictures with the most unequivocal name (cf. [16]). In order to create a picture set suitable for children, we included two-syllabic words with a consonant-vocal-onset without consonant clusters in German and an age of acquisition of 60–70 months [54]. To test whether all participants knew the words associated with the pictures, we analyzed accuracy of production of the correct picture name aloud, i.e., each missing of vocalization of the target word in the overt condition was counted as an error. Accuracy of overt picture-naming was 95.34%, indicating that the children were able to perform the task and knew almost all presented words.

### 2.3. Tasks and Procedure

The study was performed in the Lab for Cognitive Neuroscience at the Department for Hearing, Speech, and Voice Disorders of the Medical University of Innsbruck, Austria. The Ethical Committee of the Medical University of Innsbruck, Austria, granted permission to conduct this study (Ethical No. AN2016-0072 361/4.5). Methods were applied in accordance with the relevant guidelines and regulations and were in compliance with the Declaration of Helsinki. Prior to the experiment we obtained written informed consent from participants’ parents.

The picture naming task was programmed with Presentation Software (Neurobehavioral Systems, Inc., Berkeley, CA, USA, Version 18.1). Each participant sat in front of a 24′ computer monitor at a distance of 100 cm. The pictures were presented on a light grey screen. Each trial (Ø 15000 ms) started with a fixation cross (1000 ms), followed by a red speech or blue thinking bubble (2000 ms) initiating the Preparation Phase. These visual cues indicated whether the participants had to name the subsequently presented picture during the Execution Phase aloud (overt speech) or silently (inner speech). Between the preparation and execution phase the fixation cross was presented for 1000 ms. The execution phase lasted 3000 ms (Figure 1). After each picture a variable inter-stimulus-interval (ISI) with a mean duration of 8000 ms (6000–10,000 ms) showing a fixation cross followed. Because hemodynamic responses are sluggish, the variable ISI prevented a systematic overlap of the hemodynamic response of the fNIRS [55]. The selection of the duration of the preparation and execution phase was matched with the study of Kell et al. [42] and Gehrig et al. [41] where the preparation phase lasted 2000–4000 ms and the execution phase 2000–3000 ms.

During overt speech in the execution phase, participants were instructed to name the presented picture as softly as possible to reduce mouth-movement-related artifacts but loud enough to be heard and logged by the experimenter. During inner speech, participants were instructed to name the picture silently in one’s mind without any vocalization.

The pictures were presented in a mini-block design. Each mini-block contained 5 pictures of the same condition (either inner or overt speech). Totally, 80 trials (40 inner and 40 overt speech) were presented in 16 mini-blocks. Four different pseudo-randomization versions were created with maximally 4 mini-blocks of the same condition in succession.

Before EEG and fNIRS measurements, each participant performed 10 practice items (five in overt and five in inner speech) to familiarize with the task. If the subjects failed the practice items, further explanation was provided. Participants were instructed to avoid body and head movements during the experiment. The experiment lasted 20 min in total.

### 2.4. fNIRS/EEG Data Recordings

#### 2.4.1. fNIRS Data Recording

**Figure 2:**
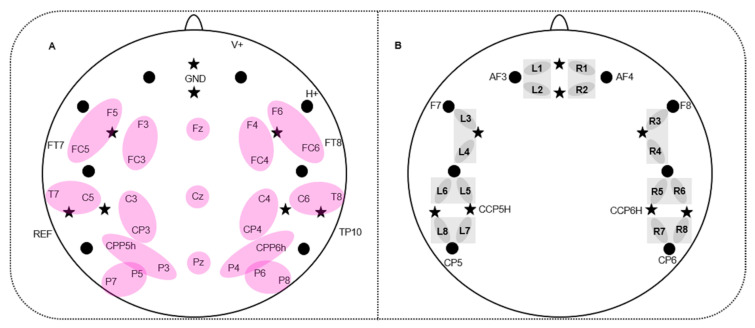
Simultaneous electroencephalography- (EEG)-electrodes and functional near-infrared spectroscopy- (fNIRS)-channel placement. (**A**) 32 EEG-electrodes and fNIRS probe arrangement; stars indicate 8 fNIRS light emitters; dots indicate 8 fNIRS detectors; purple ellipses indicate regions of interest (ROIs) of the EEG which entered statistical analyses. (**B**) fNIRS-channel placement: L1-8 show 8 left-hemispheric fNIRS-channels; R1-8 show 8 right-hemispheric fNIRS channels. fNIRS optodes which were positioned in a standard EEG electrode position are labeled, e.g., AF3. Grey squares refer to the regions of interest (ROIs) of the fNIRS channels which were used for statistical analyses.

While EEG gains an excellent temporal resolution in the range of milliseconds, fNIRS allows for a better localization of brain activity by assessing changes in the concentration of oxy-hemoglobin (oxy-Hb) and deoxy-hemoglobin (deoxy-Hb). fNIRS is a suitable soundless method for monitoring speech production and has a reduced sensitivity towards movement artifacts [56]. Physiologically, fNIRS uses neurovascular coupling to link regional blood flow and blood velocity to neural activity. An enhanced neural activation in a brain region elicits an increase in oxygen demand resulting in an increase in (oxy-Hb) and a decrease in (deoxy-Hb) [55,56,57,58]. For the present study, we used a NIRScout system (NIRxMedizintechnik GmbH, Berlin, Germany) measuring light attenuation at 760 and 850 nm in a cw-mode with a sampling rate of 7.81 Hz. Eight light emitters and eight light detectors were used resulting in 16 channels distributed over bilateral prefrontal, frontal, temporal, and parietal areas. The inter-optode-distance was 3.5 cm (e.g., [48]). Positioning of fNIRS light emitters and detectors was based on the standard EEG 10-20 and 10-5 electrode positioning system [59,60]. fNIRS optodes were integrated into a commercially available elastic EEG cap (actiCAP, Brain Products, Gilching; Germany). Recent studies in adults [61,62] and infants [63] used this electrode positioning as a reference to project to underlying anatomical structures in order to provide a better mapping of brain signals assessed from the scalp. These approaches rely on adult brains. To our knowledge, there is no similar approach considering the brain of 6–7-year-old children as in our study. To keep hairs under the optodes aside and thus ensure a good optode-skin-contact, an EEG gel was used. Head circumference of children were also quite similar (52.62 cm on average; range: 52–54 cm), thus ensuring the measurement of the same underlying brain areas. A modified EEG cap allowed for simultaneous EEG and fNIRS recordings, see Figure 2.

#### 2.4.2. EEG Data Recording

The electrophysiological signal was recorded by means of 32 active AgAgCl-electrodes placed in an elastic cap (actiCAP, Brain Products, Gilching; Germany) by using the BrainAmp EEG amplifier and Brain Vision Recorder software (Brain Products, Gilching, Germany). The electrodes were positioned at the following standardized positions [59]: F5/F3/FT7/FC5/FC3/T7/C5/C3/CPP5H/CP3/P7/P5/P3/F6/F4/FT8/FC6/FC4/T8/C6/C4/CPP6H/CP4/P8/P6/P4/Fz/Cz/Pz/F10/Fp2/TP9/TP10 and AFz (Figure 2A). From the electrode Fp2 (V+) the vertical electro-oculogram (VEOG) and from F10 (H+) the horizontal electro-oculogram (HEOG) were recorded. The ground electrode was placed at AFz. The EEG was online referenced to the left mastoid (TP9) and offline re-referenced to averaged mastoids including the left and right mastoid (TP10). Impedance of electrodes was kept below 5 kΩ. The EEG-signal was online digitized with an anti-aliasing Butterworth (slope of 24 db/Oct) band-pass filter between 0.016 Hz and 450 Hz.

### 2.5. Data Analyses

#### 2.5.1. fNIRS Data Analyses

A MATLAB (MathWorks, Inc., Natick, MA, USA, Version R2018a)-based program nilab2 (written by Stefan Paul Koch, Charité University Medicine, Berlin, Germany) was used for analyzing fNIRS data. Data were analyzed per subject and per phase (preparation, execution) based on the modified Beer-Lambert Law [64,65]. Artifacts were selected manually and corrected by a linear interpolation approach. In the next step, fNIRS data were low-pass-filtered using a third order Butterworth filter at 0.4 Hz to attenuate high frequency artifacts mainly resulting from the heart beat. A general linear model (GLM) including inner and overt speech as separate boxcar-predictors was applied using a canonical hemodynamic response function (HRF) peaking at 5 s [66]. Studies on the exact timing of the HRF peak during development provide a mixed picture. While there is sporadic evidence for faster peaks in newborns compared to adults with a peak latency of 2–4 s [67], the majority of studies in newborns showed a slower hemodynamic response compared to adults with a peak latency of 6–8 s [68,69] and 12–16 s [70] after stimulus onset. Furthermore, these studies revealed that with increasing age the peak latency of the hemodynamic response decreases. Studies in preschool children aged around 4–11 years showed similar peak latencies as adults with a peak latency between 3–8 s [71,72]. These results suggest that applying a HRF with a peak at 5 s in 6- to 7-year old children as investigated in our study might be adequate. During this modelling, a stimulation period of 2 s for the preparation phase and of 3 s for the execution phase (i.e., on-condition) and a resting period (i.e., off-condition; silence) resulting from ISIs was assumed and a high-pass filter of 30 s to remove drifts and slow fluctuations was applied. GLM using a canonical HRF (peak at 5 s and further 15 s for return to baseline) provides Beta-values for each condition (inner/overt), each channel, and each hemoglobin (oxy, deoxy) which were used for statistical analyses. Finally, fNIRS data were averaged across participants.

Statistical analyses for (oxy-Hb) and (deoxy-Hb) were carried out over 8 regions of interest (ROIs) (4 left and 4 right-hemispheric ROIs): PREF: prefrontal (L1/L2; R1/R2; L = left; R = right); FRONT: frontal (L3/L4; R3/R4); TEMP: temporal (L5/L6; R5/R6) and TPAR: temporo-parietal (L7/L8; R7/R8) (see Figure 2B).

Four-factorial repeated measure ANOVAs (CONDITION*PHASE*REGION*HEMISPHERE) were performed for (oxy-Hb) and (deoxy-Hb), separately. The ANOVAs included the within-subject factors CONDITION (overt versus inner speech), PHASE (preparation versus execution), REGION (PREF versus FRONT versus TEMP versus TPAR), and HEMISPHERE (left versus right). Please note that the combination of the factors REGION and HEMISPHERE result in the analyzed ROIs. Corrected significance according to Greenhouse and Geisser [73] was applied whenever the degrees of freedom exceeded 1. Post-hoc *t*-tests were performed using Bonferroni correction whenever the interaction between CONDITION with PHASE and/or REGION and/or HEMISPHERE reached significance.

#### 2.5.2. EEG Data Analyses

The Brain Vision Analyzer 2 (Brain Products, Gilching, Germany) software was used for analyzing EEG data. First, a 30-Hz low-pass Butterworth zero-phase filter (slope: 12 dB/oct) was applied offline. EEG data were segmented into 1200 ms epochs from −200 ms to 1000 ms, where 0 ms represents the picture onset. Before averaging, ocular correction [74] and manual artefact rejection were applied. Participants were excluded from statistical analyses when more than 15 items per condition (overt/inner) and per phase (Preparation, Execution) for at least half of electrodes were contaminated by artifacts. Luckily, no subject had to be excluded as this criterion was not satisfied. In total, 81.15% of trials survived artifact rejection for overt speech in the preparation phase, 81.65% for inner speech in the preparation phase, 82.08% of overt speech in the execution phase and 82.33% for inner speech in the execution phase. Next, data were then re-referenced to averaged mastoids. Further, a pre-stimulus baseline correction of −200 ms (0 ms represents the stimulus onset) was applied. Afterwards, data were averaged per electrode, participant, condition, and phase. Finally, a grand-average across participants was performed without the application of any further low-pass filter for presentation purposes (see Appendix A Figure A4, Figure A5 and Figure A6).

Based on a 50-ms analysis, in which paired-sample *t*-tests between overt and inner speech, separately for the preparation and execution phase, were performed from 100 to 600 ms in 50 ms consecutive segments, as well as on the literature [16,23,24,75,76], 4 time windows were analyzed (see Appendix A Table A1 for detailed information): 100–200 ms, 200–300 ms, 300–500, and 500–600 ms. After 600 ms no time window was defined because articulatory processes are found to start after 600 ms [23].

Statistical analyses were performed on 12 regions of interest (ROI) over the left and right hemisphere including two electrodes each: left: F3-FC3, F5-FC5, C3-CP3, C5-T7, CPP5H-P3, P5-P7, right: F4-FC4, F6-FC6, C4-CP4, C6-T8, CPP6H-P4, P6-P8. Additionally, the three midline electrodes (Fz, Cz and Pz) were analyzed separately.

In analogy to fNIRS, repeated measure ANOVAs (CONDITION*PHASE*REGION*HEMISPHERE) were performed for the 4 selected time windows, separately. These ANOVAs for lateral electrodes comprised the within-subject factors CONDITION (overt versus inner speech), PHASE (preparation versus execution), REGION (6 lateral ROIs), and HEMISPHERE (left versus right). The ANOVAs for midline electrodes included the within-subject factors CONDITION, PHASE, and REGION (Fz versus Cz versus Pz). Significance level was assumed at *p* < 0.05. Whenever the interaction between CONDITION with PHASE and/or REGION and/or HEMISPHERE reached significance, post-hoc *t*-tests were performed applying a Bonferroni correction. Corrected significance according to Greenhouse and Geisser [73] was applied whenever the degrees of freedom exceeded 1.

## 3. Results

### 3.1. fNIRS Results

**Figure A1:**
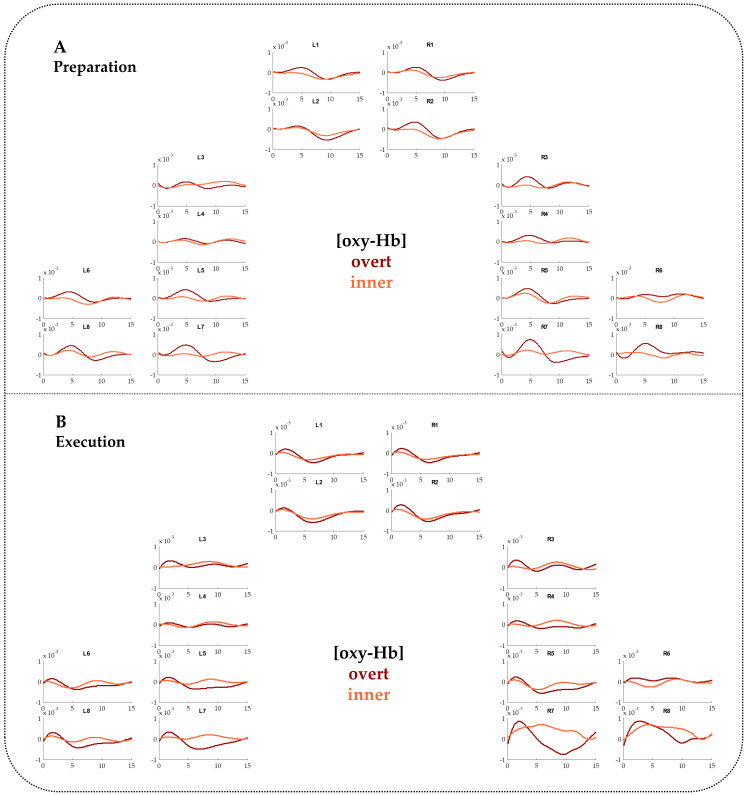
FNIRS time courses for [oxy-Hb] contrasting overt and inner speech for the Preparation phase (**A**) and the Execution phase (**B**).

**Figure A2:**
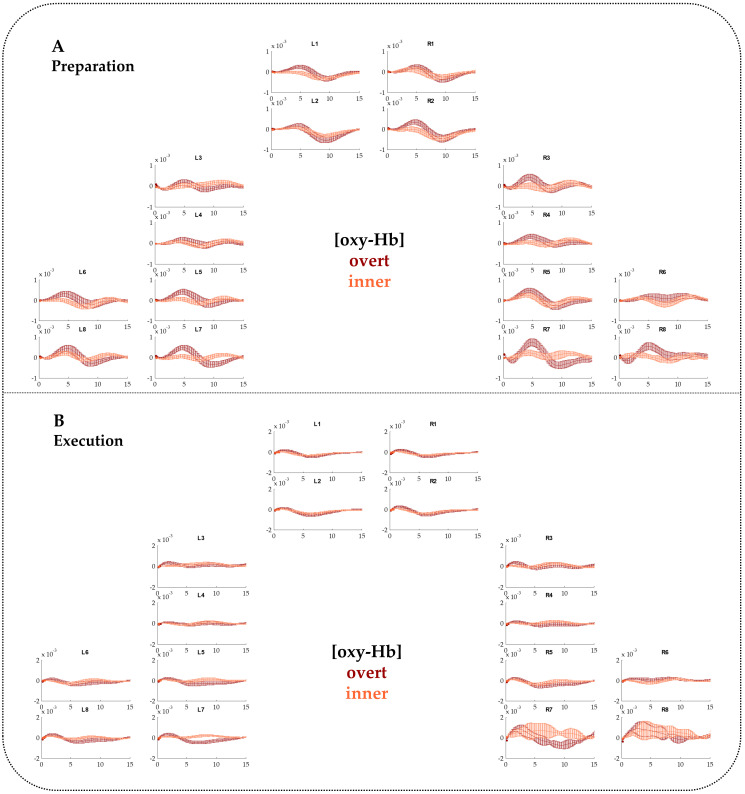
FNIRS time courses including standard errors of the mean (SEMs) for [oxy-Hb] contrasting overt and inner speech for the Preparation phase (**A**) and the Execution phase (**B**).

*Oxy-Hb.* The repeated measure ANOVA revealed a significant interaction CONDITION*PHASE (*F*_(1,28)_ = 4.36, *p* = 0.046, *ŋ_p_^2^* = 0.135). Post-hoc *t*-tests yielded no significant differences (see Appendix A Figure A1 and Figure A2 for fNIRS time courses with and without standard errors of the mean (SEMs)).

**Figure 3:**
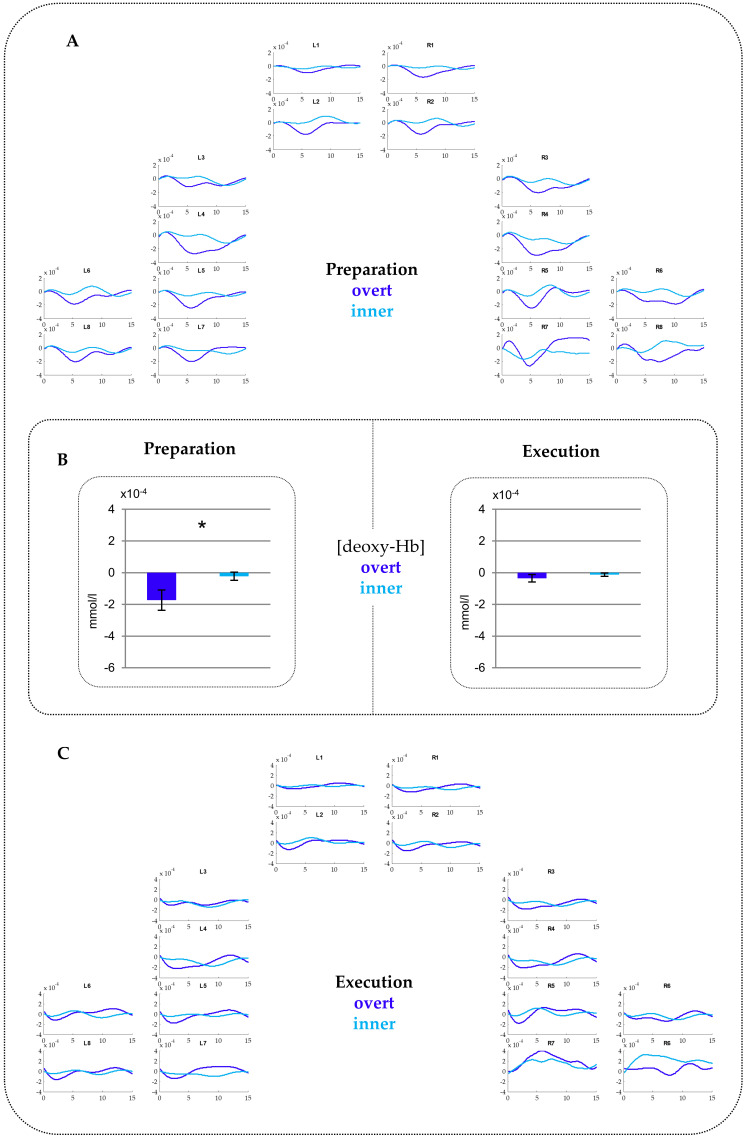
fNIRS results for (deoxy-Hb). (**A**) Time courses for the Preparation phase. (**B**) Beta-values comparing overt and inner speech for the Preparation phase (left) and Execution phase (right) merged over all ROIs. The asterisk indicates statistically significant differences between overt versus inner speech. Please note that a more negative value for (deoxy-Hb) indicates increased activations. (**C**) Time courses for the Execution phase. See Appendix A Figure A3 for time courses for the Preparation and Execution phase in (deoxy-Hb) including standard errors of the mean (SEMs).

*Deoxy-Hb.* The ANOVA showed a significant main effect of CONDITION (*F*_(1,28)_ = 7.16, *p* = 0.012, *ŋ_p_^2^* = 0.204) as well as a significant interaction CONDITION*PHASE (*F*_(1,28)_ = 4.42, *p* = 0.045, *ŋ_p_^2^* = 0.136). Post-hoc testing resolving the interaction indicated a larger activation for overt compared to inner speech during the preparation phase (*t*_(28)_ = −2.53, *p* = 0.017). Notably, this activation extended over all ROIs as no interaction with region and/or hemisphere were statistically ascertained. No difference between overt and inner speech was found for the execution phase. Figure 3 provides time courses (A and C) for each channel and beta-values merged over all ROIs (B). This shows the comparison between overt versus inner speech in the preparation and execution phase, separately.

### 3.2. EEG Results

**Figure A4:**
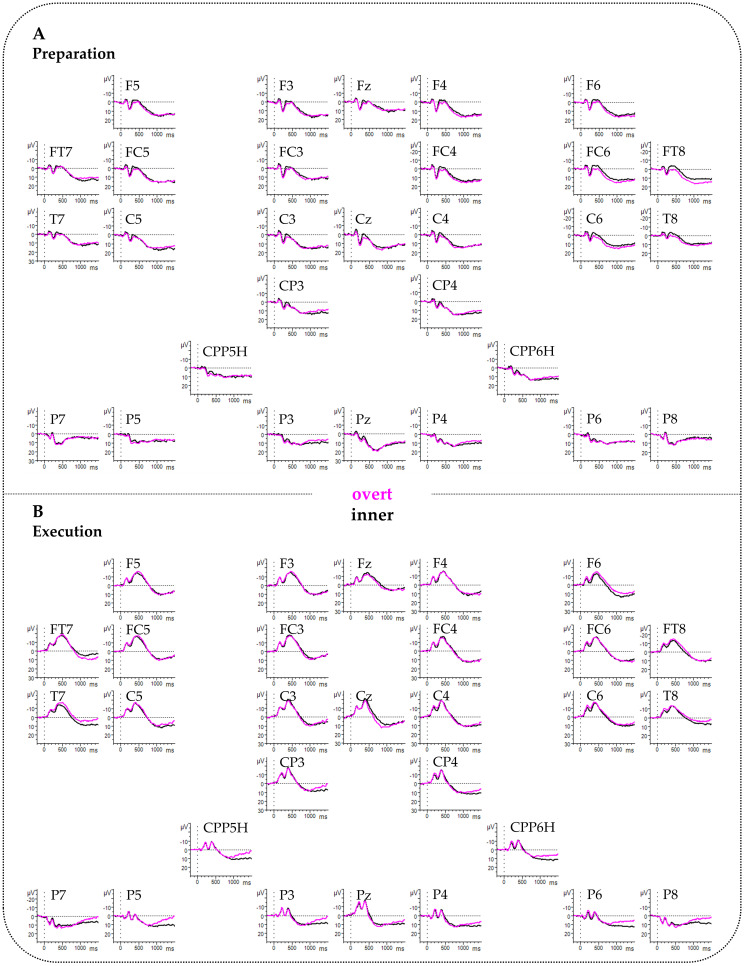
Event-related brain potentials (ERPs). (**A**) Grand averages for the Preparation phase. (**B**) Grand averages for the Execution phase. Negative polarity is plotted upward. The x-axis ranges from −200 to 1500 ms. No low-pass filter was applied for presentation purposes.

**Figure A5:**
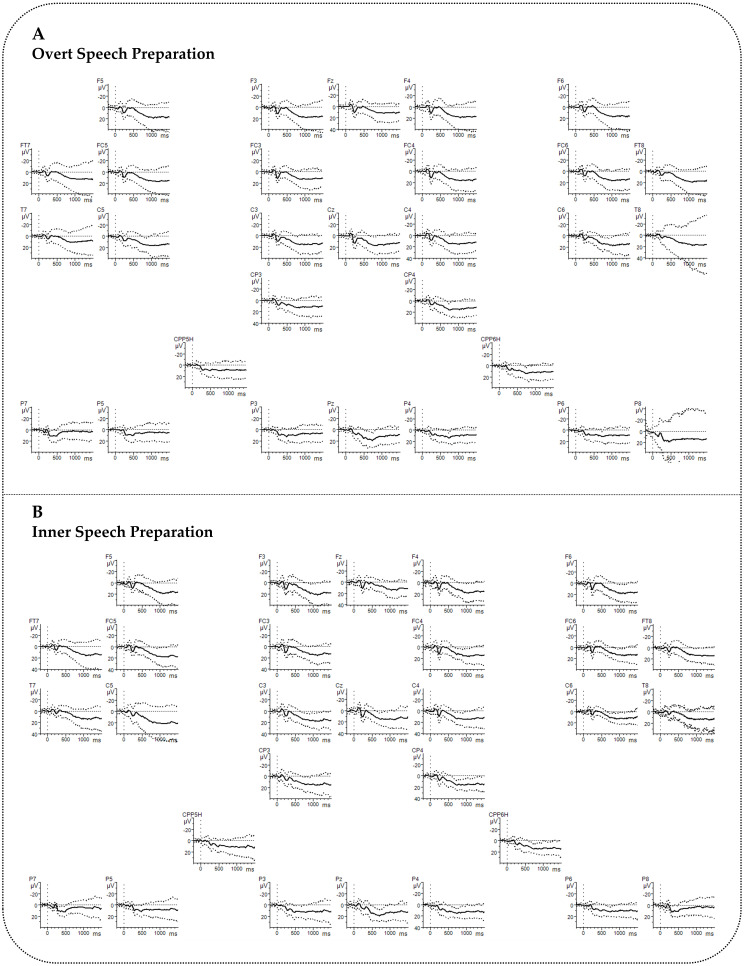
Event-related brain potentials (ERPs) for the Preparation phase including standard deviations. (**A**) Grand averages for the Overt Speech Preparation (**B**) Grand averages for the Inner Speech Preparation. Negative polarity is plotted upward. The x-axis ranges from −200 to 1500 ms. No low-pass filter was applied for presentation purposes.

**Figure A6:**
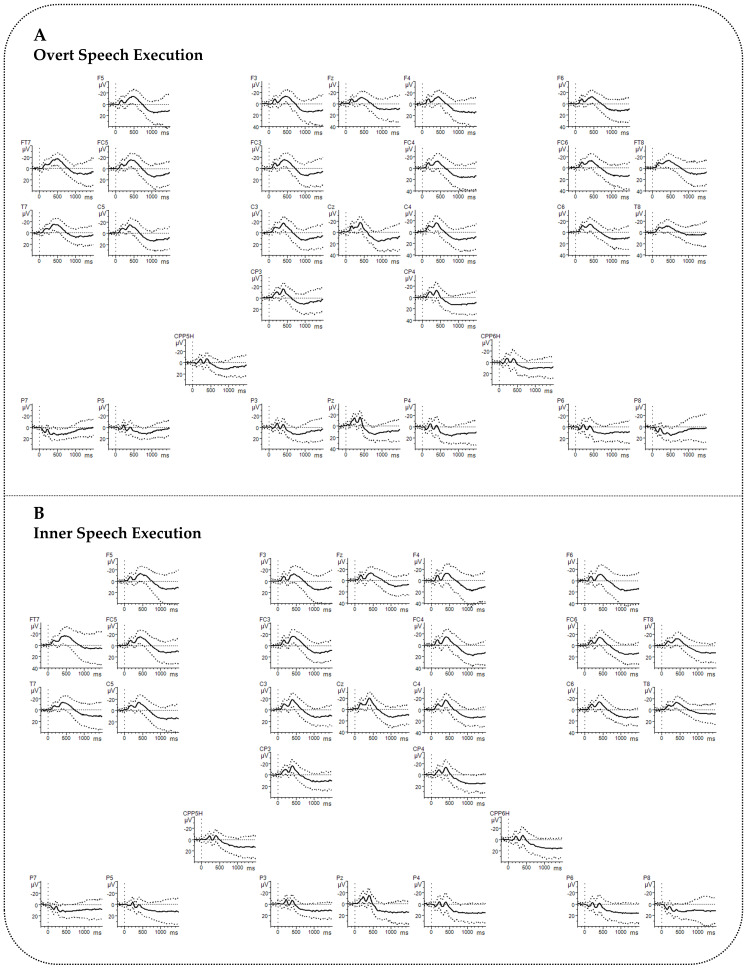
Event-related brain potentials (ERP) for the Execution phase including standard deviations. (**A**) Grand averages for the Overt Speech Execution (**B**) Grand averages for the Inner Speech Execution. Negative polarity is plotted upward. The x-axis ranges from −200 to 1500 ms. No low-pass filter was applied for presentation purposes.

The ANOVAs for the time windows 100–200 ms, 200–300 ms, and 500–600 ms did not show any significant effect. For the time window 300–500 ms a significant interaction CONDITION*REGION (*F*_(2,50)_ = 4.22, *p* = 0.030, *ŋ_p_^2^* = 0.144) was present at midline electrodes. However, post-hoc testing did not survive Bonferroni correction. See Appendix A Figure A4, Figure A5 and Figure A6 for ERPs with and without standard deviations for the preparation and the execution phase.

## 4. Discussion

The present study aimed at examining inner and overt speech during speech execution as well as during the setting up of the language network in children to disentangle domain-general executive control from more linguistically driven processes. To reach this goal participants completed a picture naming task by naming the pictures aloud (overt speech) or silently (inner speech). A neutral cue (a speech or thinking bubble) during the preparation phase (measuring the setting up of the language network) indicated whether the participants had to name the subsequently presented picture during the execution phase (measuring speech production per se) aloud or silently. We simultaneously measured the electroencephalography (EEG), event-related brain potentials (ERPs) in particular, and the functional near-infrared spectroscopy (fNIRS) to identify fast dynamic mechanisms and involved brain areas.

### 4.1. Differences Between Overt and Inner Speech Preparation

FNIRS results in our children revealed a larger activation for overt compared to inner speech over prefrontal, frontal, temporal, and parietal regions within the preparation phase only. Stephan et al. [16], using the same experimental design as in the present study in adult participants, also found a larger activation for overt compared to inner speech over prefrontal to parietal regions during a preparation phase and over temporal regions during the execution phase. Especially, the activation found in our children during the preparation phase largely corresponds in direction of effects and topography to that of adults. Previous studies focusing on the execution phase showed largely overlapping activated brain regions between children and adults [33,34,35,36]. Similar studies including a preparation phase are not available so far. Our results show that children predominantly use the same brain regions as adults in the preparation of subsequent language production processes. Generally, a larger activation for overt compared to inner speech in prefrontal and frontal regions was assumed to reflect motor planning during phonological and lexical processing [77] while temporal regions have been proposed to reflect monitoring mechanisms of motor output such as auditory feedback control [56,78,79]. However, the fMRI studies leading to these findings directly focused on speech execution and did not integrate a preparation phase. As already introduced, our task design is based on the designs of Gehrig and colleagues [41] as well as Kell and colleagues [42] who related processes during the preparation phase to executive control (i.e., mechanisms for controlling the selection and implementation of selected rules to be turned into goal-directed behavior) rather than to specific linguistic processes as the preparation phase does not include any concrete linguistic or motor process. Both studies [41,42] found a widespread bilateral activation over prefrontal and perisylvian areas as well as left planum temporale suggesting that the brain prepares the executive system in anticipation of the behavioral control of the planned action. The authors stated that the involvement of these regions, especially Broca, reflects increased demands for executive control. The activation of the left planum temporale was assumed to index the preparation of the sensory system for a subsequent auditory feedback after overt speech execution. Thus, these studies showed that before speech is acted out and articulation is planned, the brain prepares for the sensory and motor consequences of speaking. Generally, the bilateral fronto-temporo-parietal network has been identified to be involved during goal-directed behavior, selective attention, and response inhibition that require domain-general (nonlinguistic) executive control [80,81,82,83,84,85,86,87,88,89]. Furthermore, this network was found to be associated with the sensorimotor adaption for speech [90,91,92]. In our experiment, to prepare overt articulation by keeping in mind minimization of mouth movements, and controlling the voice amplitude such that the subsequently produced word is loud enough but not too loud, might have imposed a greater task demand than the inner speech condition. Thus, a higher activation for overt compared to inner speech might represent increased attentional and inhibitory control processes [90,91,92]. Geranmayeh et al. [45] stated that the left fronto-temporo-parietal network incorporates and controls lexical, phonological, and syntactical expressions requiring linguistic control (i.e., domain-specific executive control), whereas the right fronto-temporo-parietal network is engaged in domain-general executive processes. Thus, the bilateral topography of our fNIRS results during the preparation phase might resemble the involvement of both domain-general and domain-specific control mechanisms [16,41,42]. Inner speech might be less activated than overt speech because inner speech does not need to prepare subsequent speech-related processes (e.g., the preparation for subsequent phonological retrieval and encoding but also the control for mouth movements and loudness adjustments) to the same extent as overt speech [23]. Thus, our findings in children seem to indicate also that the brain prepares the subsequent speech execution differentially for inner and overt speech, yet in a similar way as in adults. We suggest that already the children’s brain can differentiate between inner and overt speech by exerting different executive control for the subsequent inner and overt speech execution.

Interestingly, the EEG did not show any significant difference between inner and overt speech during the preparation phase while the fNIRS did. This is in contrast to the adult data which showed a larger negativity for inner compared to overt speech from 200 up to 500 ms in the EEG [16]. The adult results during the preparation phase indicate that the speech production network pre-activates and pre-inhibits relevant processes in anticipation of the linguistic processes necessary for the execution of overt and inner speech. That means the brain selects and implements appropriate rules for behavioral control during the execution phase. Indeed, this became evident for the adult data, showing a larger negativity for inner compared to overt speech also during the execution phase. This indicates the presence of executive control processes including inhibition. Previous EEG studies which specifically investigated inhibitory processes in preschool children found a larger N200 for no-go stimuli compared to go-stimuli [93,94]. This larger N200 was interpreted as greater inhibitory control for rarely presented no-go stimuli which have to be suppressed with respect to the motor response. This pattern resembles that of adults and demonstrates that children are able to inhibit certain kind of information. It should be noted that a motor go-/no-go task and our inner-overt speech task do not completely correspond but both capture inhibitory mechanisms. The fact that our fNIRS results showed differential processing between inner and overt speech during the preparation phase also indicates such an ability in 6–7-year-old children. Thus, an explanation of why the EEG did not result in any difference unfortunately remains speculative. The electrophysiological signal measures more temporally fine-grained and fast dynamic processes while fNIRS assesses a slower vascular response. Potentially, the fNIRS seems to be more sensitive to capture these slow changes within a speech preparation paradigm in children. We thus believe that the child’s brain needs more time to set up the speech production network than the adult brain. However, it remains speculative whether setting up the language network is more challenging for children and thus might lead to slower neural responses. This emphasizes the importance of a multi-methodological approach combining several methods, as in a study merely using EEG these missing differences might have been erroneously interpreted as an absent differentiation in children, while this ability could indeed be attested by the method of fNIRS. Further research needs to clarify the reasons behind the differential sensitivities of EEG and fNIRS in 6–7-year-olds.

### 4.2. Differences between Overt and Inner Speech Execution

In order to better define at which concrete speech production processing stage in time differences between inner and overt speech occur, the EEG is the method of choice. Unfortunately, the EEG results did not show any difference between inner and overt speech during the execution phase. This is in contrast to the adult data of Stephan et al. [16], who found a larger negativity for inner compared to overt speech from 300 ms up to 500 ms during the execution phase. Because this time range corresponds to phonological code retrieval and encoding in the speech production model by Indefrey [23], the authors interpreted this increased negativity for inner speech as reflecting inhibitory mechanisms at this linguistic stage. The lack of difference between inner and overt speech in the execution phase in the EEG for children might account for the unimpoverished hypothesis [18,19]. This hypothesis proposes that inner and overt speech do not differ at the phonological level. In order to definitely prove whether these mechanisms are the driving force in our 6–7-year-old children, certainly more studies are needed. However, if the unimpoverished hypothesis is true and potentially represents a rather immature processing of inner speech in school-aged children, our results might also explain why the development of inner speech can be accompanied by external manifestations like whispering, muttering or lip moving [6,7,8]. Indeed, in our experiment some of these external manifestations were also attested during the inner speech condition, even though they represented a rather small portion (3.1%). Nevertheless, the presence of some external manifestations together with a missing neural differentiation between inner and overt speech allows speculation that the neural underpinnings of inner and overt speech in 6–7-year-old children might still be not fully developed yet. Of course, further studies with varying experimental designs and subjects of different age ranges are necessary to definitely support this claim.

With respect to topographical aspects, during the execution phase per se no differences between inner and overt speech were found in children. This is in contrast to adults who showed a larger activation for overt compared to inner speech over bilateral temporal regions [16]. In adults, this activation was interpreted as reflecting auditory feedback mechanisms, i.e., the auditory perception of one’s own speech. Interestingly, 6–7-year-old children in the presented study did not show differences between inner and overt speech during the execution phase. Krishnan et al. [36] also revealed similar findings. They found a larger bilateral temporal activation during an overt picture-naming task in adults in contrast to children. This result was interpreted in such a way that adults rely more on auditory feedback mechanisms and online correction of spoken output than children. A behavioral study proposed that, while 9–11-year-old children are able to compensate for auditory feedback perturbations during speech motor output, they do not show a reliable compensatory effect on their perceptual representations [95]. The authors suggested that perceptual abilities in 9–11-year-old children are not fully adult-like. Thus, our results might follow this line of reasoning in that children seem to be not fully able to use complex auditory feedback mechanisms as adults do, suggesting immature processes. Although it seems that children are able to prepare different sensory and motor consequences of speaking (indexed by differences in domain-general executive control in the preparation phase), we carefully suggest an immature neural processing for feedforward auditory prediction and online correction of inner and overt speech production (i.e., domain-specific executive control) in children. It therefore could be possible that children do not adapt their phonological processes for inner and overt speech in the same way as adults do [36,95]. Definitely, further research is required in order to investigate auditory feedback in overt and inner speech in children in a more detailed fashion. Taken together, our findings suggest that the children’s brain sets up the speech production network for overt and inner speech differentially by requiring different domain-general executive control processes. However, the children’s brain cannot differentiate between inner and overt speech when a concrete linguistic content occurs and a concrete speech execution is required. Potentially, this indicates immature phonological code retrieval and encoding, auditory feedback, and domain-specific executive control processes.

## 5. Conclusions

The present study provides some evidence that children use similar brain regions as adults to prepare inner and overt speech. When linguistic aspects become relevant, as in the speech execution phase, children do not seem to be fully able to use phonological encoding as well as complex auditory feedback mechanisms as well as adults. More specifically, the missing difference between inner and overt speech during the execution phase might support the unimpoverished hypothesis. Thus, the internalization process in 6- to 7-year-old children seems to be still developing neurally towards becoming completely inner speech [4,96]. Further research should shed more detailed light on the relation between this age-dependent increase of inner-overt distinction and the relation to cognitive processes.

## Figures and Tables

**Figure A3:**
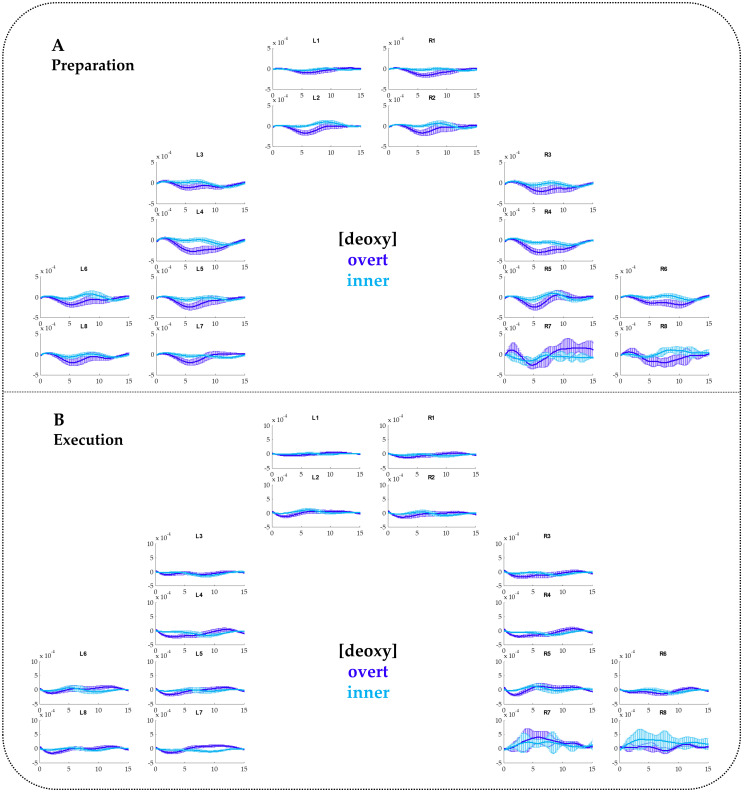
FNIRS time courses for [deoxy-Hb] including standard errors of the mean (SEMs) contrasting overt and inner speech for the Preparation phase (**A**) and the Execution phase (**B**).

## Appendix A

**Table A1:** EEG Results of the 50 ms analysis. Paired-sample *t*-tests were performed between inner and overt speech, separately for the preparation and execution phase between 100–600 ms in 50 ms consecutive steps; numbers indicate significant *p*-values; ns indicates non-significant.

**Preparation**										
**ms**	**100–150**	**150–200**	**200–250**	**250–300**	**300–350**	**350–400**	**400–450**	**450–500**	**500–550**	**550–600**
**Electrodes**										
F3	ns	ns	ns	ns	ns	ns	ns	ns	ns	ns
F5	ns	ns	ns	ns	ns	ns	ns	ns	ns	ns
FC3	ns	ns	ns	ns	ns	ns	ns	ns	ns	ns
FC5	ns	ns	ns	ns	ns	ns	ns	ns	ns	ns
TF7	ns	ns	ns	ns	ns	ns	ns	ns	ns	ns
C3	ns	ns	ns	ns	ns	ns	ns	ns	ns	ns
C5	ns	ns	ns	ns	ns	ns	ns	ns	ns	ns
T7	ns	ns	ns	ns	ns	ns	ns	ns	ns	ns
CP3	ns	ns	ns	ns	ns	ns	ns	ns	ns	ns
CPP5H	ns	ns	ns	ns	ns	ns	ns	ns	ns	ns
P3	ns	ns	ns	ns	ns	ns	ns	ns	ns	ns
P5	ns	ns	ns	ns	ns	ns	ns	ns	ns	ns
P7	ns	ns	ns	ns	ns	ns	ns	ns	ns	ns
F4	ns	ns	ns	ns	ns	ns	ns	ns	ns	ns
F6	ns	ns	ns	ns	ns	ns	ns	ns	ns	ns
FC4	ns	ns	ns	ns	ns	ns	ns	ns	ns	ns
FC6	ns	ns	ns	ns	0.039	ns	ns	ns	ns	ns
FT8	ns	ns	ns	0.006	0.001	0.006	0.006	0.011	0.050	0.034
C4	ns	ns	ns	ns	0.018	ns	ns	ns	ns	ns
C6	ns	ns	ns	0.014	0.020	ns	ns	ns	ns	ns
T8	ns	ns	ns	ns	ns	ns	ns	ns	ns	ns
CP4	ns	ns	ns	ns	ns	ns	ns	ns	ns	ns
CPP6H	ns	ns	ns	ns	ns	ns	ns	ns	ns	ns
P4	ns	ns	ns	ns	ns	ns	ns	ns	ns	ns
P6	ns	ns	ns	ns	ns	ns	ns	ns	ns	ns
P8	ns	ns	ns	ns	ns	ns	ns	ns	ns	ns
Fz	ns	ns	ns	ns	ns	ns	ns	ns	ns	ns
Cz	ns	ns	ns	0.010	0.002	ns	ns	ns	ns	ns
Pz	ns	ns	ns	ns	ns	ns	ns	ns	ns	ns
**Execution**										
**ms**	**100–150**	**150–200**	**200–250**	**250–300**	**300–350**	**350–400**	**400–450**	**450–500**	**500–550**	**550–600**
**Electrodes**										
F3	ns	ns	ns	0.098	ns	ns	ns	ns	ns	ns
F5	ns	ns	ns	ns	ns	ns	ns	ns	ns	ns
FC3	ns	ns	ns	ns	ns	ns	ns	ns	ns	ns
FC5	ns	ns	ns	ns	ns	ns	ns	ns	ns	ns
TF7	ns	ns	ns	ns	ns	ns	ns	ns	ns	ns
C3	ns	ns	ns	ns	ns	ns	ns	ns	ns	ns
C5	ns	ns	ns	ns	ns	ns	ns	ns	ns	ns
T7	ns	ns	ns	0.036	ns	ns	ns	ns	ns	ns
CP3	ns	ns	ns	ns	ns	ns	ns	ns	ns	ns
CPP5H	ns	ns	ns	ns	ns	ns	ns	ns	ns	ns
P3	ns	ns	ns	ns	ns	ns	ns	0.038	ns	ns
P5	ns	ns	ns	ns	ns	ns	ns	ns	ns	ns
P7	ns	ns	ns	ns	ns	ns	ns	ns	ns	ns
F4	ns	ns	ns	ns	ns	ns	ns	ns	ns	ns
F6	ns	ns	0.014	0.001	ns	ns	ns	ns	ns	ns
FC4	ns	ns	ns	ns	ns	ns	ns	ns	ns	ns
FC6	ns	ns	0.034	0.007	ns	ns	ns	ns	ns	ns
FT8	ns	ns	0.019	0.035	ns	ns	ns	ns	ns	ns
C4	ns	ns	0.007	0.010	ns	ns	ns	ns	ns	ns
C6	ns	0.029	0.001	0.001	ns	ns	ns	ns	ns	ns
T8	ns	ns	0.036	ns	ns	ns	ns	ns	ns	ns
CP4	ns	ns	ns	ns	ns	ns	ns	ns	ns	ns
CPP6H	ns	ns	ns	ns	ns	ns	ns	ns	ns	ns
P4	ns	ns	ns	ns	ns	ns	ns	0.029	0.035	ns
P6	ns	ns	ns	ns	ns	ns	ns	ns	ns	ns
P8	ns	ns	ns	ns	ns	ns	ns	ns	ns	ns
Fz	ns	ns	ns	ns	ns	ns	ns	ns	ns	ns
Cz	ns	ns	ns	ns	ns	ns	ns	0.022	0.012	0.021
Pz	ns	ns	ns	ns	ns	ns	ns	ns	ns	ns
